# Quality assessment of randomized controlled trial abstracts on drug therapy of periodontal disease from the abstracts published in dental Science Citation Indexed journals in the last ten years

**DOI:** 10.4317/medoral.23647

**Published:** 2020-05-10

**Authors:** Lingzhi Xie, Wenguang Qin, Ting Yu, Janak L. Pathak, Sujuan Zeng, Minquan Du

**Affiliations:** 1 MOST KLOS & KLOBM, School and Hospital of Stomatology, Wuhan University, Wuhan, China; 2 Key Laboratory of Oral Medicine, Guangzhou Institute of Oral Disease, Affiliated Stomatology Hospital of Guangzhou Medical University, Guangzhou, China

## Abstract

**Background:**

Randomized controlled trials (RCTs) provide the highest level of evidence and are likely to influence clinical decision-making. This study evaluated the reporting quality of RCT abstracts on drug therapy of periodontal disease and assessed the associated factors.

**Material and Methods:**

The Pubmed database was searched for periodontal RCTs published in Science Citation Indexed (SCI) dental journals from 2010/01/01 to 2019/07/17. Information was extracted from the abstracts according to a modified Consolidated Standards of Reporting Trials (CONSORT) guideline checklist. The data was analyzed using descriptive statistical analysis and the statistical associations were examined using the linear regression analysis (*P* <0.05).

**Results:**

This study retrieved 1715 articles and 249 of them were finally included. The average overall CONSORT score was 15.6 ± 3.4, which represented 40.9% (±0.6) of CONSORT criteria filling. The reporting rate of some items (trial design, numbers analyzed, confidence intervals, intention-to-treat analysis or per-protocol analysis, harms, registration) was less than 30%. The adequate reporting rate of some items (participants, randomization, numbers analyzed, confidence intervals, intention-to-treat analysis or per protocol analysis) was no more than 4%. None of the abstracts reported funding. According to the multivariable linear regression results, number of authors (*P*=0.030), word count (*P* <0.001), continent (*P*=0.003), structured format (*P* <0.001), type of periodontal disease (*P* <0.001) and international collaboration (*P*=0.023) have a significant association with reporting quality.

**Conclusions:**

The quality of RCT abstracts on drug therapy of periodontal disease in SCI dental journals remained suboptimal. More efforts should be made to improve RCT abstracts reporting quality.

** Key words:**Abstracts, RCT, drug therapy, periodontal disease, CONSORT, reporting quality assessment.

## Introduction

Randomized controlled trials (RCTs) are magnificent in medical research and provide the highest level of evidence (1). For conducting good quality RCTs to obtain the precise clinical outcomes, appropriate design and execution are essential. Readers often screen the title and abstract of a study to decide whether to retrieve the full text or not. So abstract is a very important component of a study and is most readily available to all researchers and clinicians. Sometimes abstract may be the only information available to readers. This may be because of articles not for free, or download limitation for low Internet speed. Especially when articles published in other languages, only abstracts are translated into the readers’ own language. Hence, the accuracy and reporting quality of abstracts are considered to be of particular importance. To improve the reporting quality of RCT abstracts, guidance on reporting RCT abstracts has been released by the Consolidated Standards of Reporting Trials (CONSORT) group in 2008 (2). The CONSORT guidelines have been included in the author’s guidelines in various dental journals, and authors are required to follow the CONSORT checklist (3).

The RCT abstracts guidelines direct authors on the right way to present the information and illuminate the key points that should be included. These guidelines also recommended the use of structured format in the RCT abstracts. It has been recently reported that the quality of RCT abstracts remains suboptimal (4). Furthermore, the quality of RCT abstracts in leading dentistry journals has been documented to be suboptimal (5). A number of RCTs may have been overlooked, in which these were carried out adequately, but reported poorly. Therefore, a high level of reporting quality of RCT abstracts is as important as making a huge effort to design and conduct researches (6).

Evidence-based dentistry requires clinicians to support decisions through clinical evidence. It has been estimated that more than one hundred periodontology RCTs published every year, which are likely to impact clinical decision making. In recent years, there were some studies on the reporting quality of periodontology RCTs. In 2016, Leow NM compared the reporting quality of periodontology RCTs with the RCTs 14 years ago and showed us that the reporting quality had improved but not yet optimal (7). Satish Kumar assessed the reporting quality of periodontal RCT abstracts published in 2012 and found that substantial effort is needed to improve the reporting quality (8). Periodontal disease is a highly prevalent infectious oral disease. Periodontitis, a common periodontal disease, is highly associated with systemic consequences, including pulmonary, cardiac, and neuronal abnormalities. Therefore, the optimization of effective drug therapy for periodontal diseases is crucial to prevent systemic diseases. Drug therapy is an essential additional method of basic periodontal treatment and surgical treatment. To avoid the abuse of the drug and assure rational drug use, clinical drug therapy should be based on the current best scientific evidence and considered whether to use drug therapy and choose the appropriate drug. As RCTs are the highest level of evidence, it is imperative to evaluate the reporting quality of periodontal RCTs on drug therapy, especially their abstracts. As far as we know, this is the first time to assess the reporting of RCT abstracts on drug therapy of periodontal disease. So the aim of the present study was to evaluate the reporting quality of RCT abstracts on drug therapy of periodontal disease. In addition, the association between reporting quality and some possible factors has been identified.

## Material and Methods

- Database search strategies

The PubMed database was searched for abstracts of periodontal RCTs using the international standard serial numbers (ISSNs) of all dental Science Citation Index (SCI) journals (a total 90), according to the 2018 Journal of Citation Reports (JCR). The search was limited to “humans”, and published in the English language during the period of January 1, 2010 to July 17, 2019. The Medline Subject Heading (MeSH) terms used were “periodont* OR gingiv* NOT *implant” NOT prevent*[tiab] (9). The search strategy of RCT was “randomized controlled trial [pt] OR controlled clinical trial [pt] OR randomized [tiab] OR placebo [tiab] OR clinical trials as topic [mesh: noexp] OR randomly [tiab] OR trial [ti]”, as defined by the “Cochrane Handbook for Systematic Reviews for Interventions”. The retrieval was done on 2019-07-17.

- Inclusion criteria 

RCTs on drug therapy of periodontal disease were selected. The research reports based on laboratory trials using extracted teeth and other types, such as observational studies, cross-sectional studies, retrospective studies, case-controlled studies, reviews, meta-analysis, comments, and letters, were excluded.

- Study screening

The retrieved studies were entered into NoteExpressV3.2.0.6992. Two investigators (A. X and B. Q) independently screened the titles and abstracts of the retrieved studies. If an abstract couldn’t be identified as an RCT, its eligibility was decided following the retrieval and analysis of the full text. Any disagreement was discussed with a third investigator (C. Y).

- Data collection

Two investigators (A. X and B. Q) collected data from the included studies on general features, including category of periodontal diseases, trial outcome, drug administration, country and continent of first author, number of authors in the study, journal position in the JCR ranking, structured format, word count, international collaboration, CONSORT endorsement. The home page of CONSORT was searched to collect the information of journal CONSORT endorsement on 2019-8-10.

The reporting quality of abstracts of the included studies was assessed with a modified CONSORT checklist by two investigators (A. X and B. Q). The original CONSORT checklist for reporting RCT abstracts was designed for both journal abstracts and conference abstracts, having a total of 17 items with description (2). The present study excluded two variables, “Authors” and “Recruitment,” which were usually specific for conference abstracts. The CONSORT checklist was modified with the addition of PICO (Participants, Intervention, Comparator and Outcome) evaluation of the title, structured format and intention-to-treat analysis or per-protocol analysis, and with outcome in results break into effect estimates, confidence intervals (CI), and reporting of P-values. The final modified checklist included 19 items (Table 1).

**Table 1:**
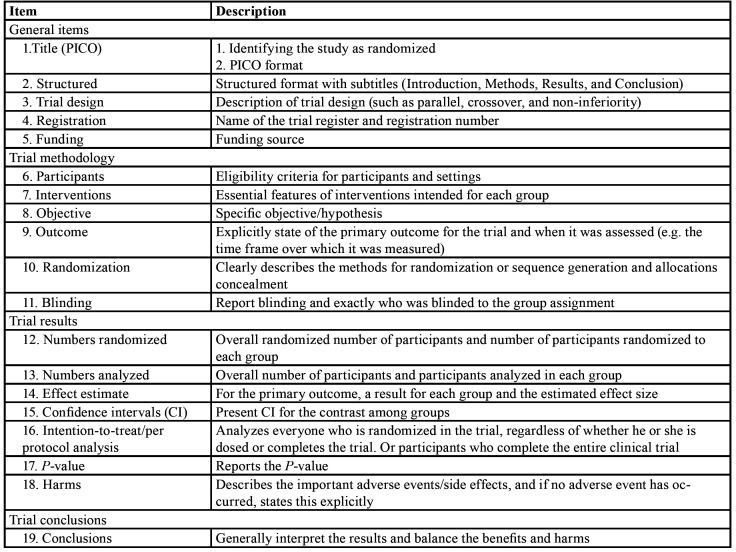
The modified checklist used for the evaluation of RCT abstracts (n=249).

The items were scored as 0, 1 and 2, corresponding to “no description”, “inadequate description” and “adequate description,” respectively. A score of 2 was considered as 100% for each variable. The overall CONSORT score (OCS) of each trial was counted including the scores of the 19 modified items. Hence, the maximum score that could be awarded to a trial was 38. In order to make the comparision to the previous studies easier, OCS% was calculated (OCS/38). All data were collected using Microsoft Excel 2007 (Microsoft Corp). Any disagreement was discussed with a third investigator (C. Y).

- Data Assessment

Data were statistically analyzed using the SPSS software (ver.23.0; IBM SPSS, USA). The mean percentage and 95% CI for each item and overall reporting quality of each RCT abstract were calculated by descriptive statistics. In addition, univariate regression analysis was conducted to evaluate the possible association between reporting quality and potential factors. Then the significant factors (*P*<0.05) were entered in multivariate regression for further analysis. For all analysis, a P-value <0.05 was considered statistically significant.

## Results

- Results of literature selection and overall CONSORT score (OQS)

**Figure 1:**
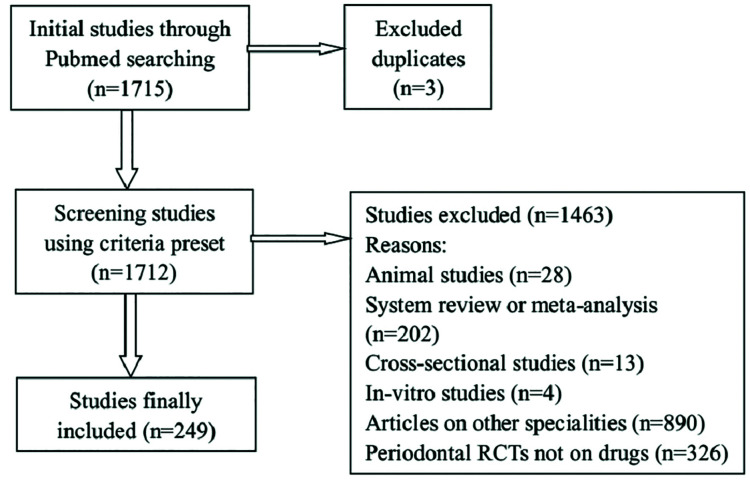
Flowchart of literature research.

As shown in Fig. 1, a total of 1715 articles were retrieved, and 249 articles that fulfilled the inclusion criteria were finally included for further analysis. The mean OQS of all 249 RCT abstracts was 15.6 ± 3.4, which represents 40.9 ± 0.6% compliance with the checklist.

- Reporting of general items

The scoring results of all 19 items are presented in Table 2. The titles of 156 (62.7%) abstracts could be identified as randomized and followed the PICO format. In totally 94.0% of the included abstracts were structured, and 88.0% of them were adequately structured. There were 70 (28.1%) abstracts described the exact trial design.

**Table 2:**
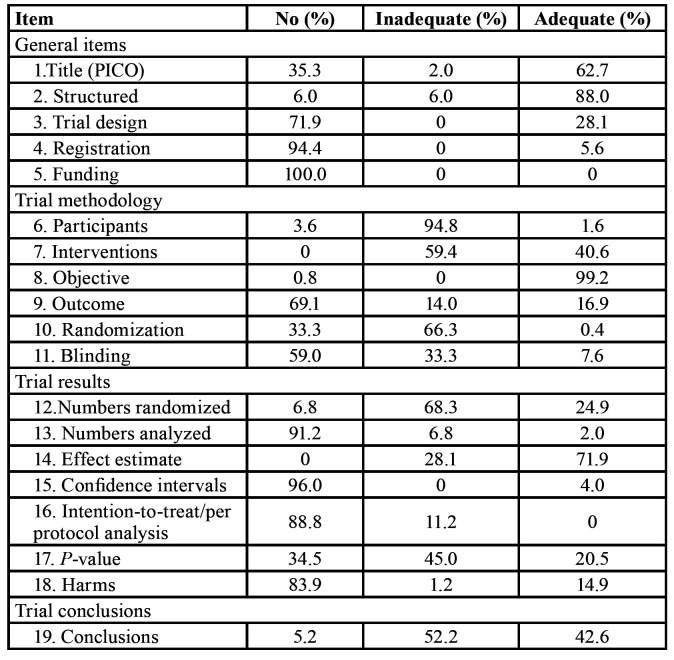
Percentage distribution of scoring for items in the modified CONSORT checklist for abstracts (n=249).

Merely 14 (5.6%) abstracts reported trial registration and none of the 249 RCT abstracts reported the funding sources.

- Reporting of trial methodology

There were 240 abstracts (96.4%) that provided the eligibility criteria for the trial participants, merely 4 abstracts (1.6%) reported the settings where the participants were investigated. All of the abstracts had the intervention description, but just 40.6% of them were reported adequately. The majority of abstracts sufficiently described the objectives (99.2%). Just 42 abstracts (16.9%) clearly stated the primary outcome. Only one abstract (0.4%) explained how the sequence generation and allocation concealment were reached. Furthermore, 102 abstracts (40.9%) mentioned blinding, but merely 19 abstracts (7.6%) accurately described who was blinded.

- Reporting of trial results

The majority of abstracts (93.2%) mentioned the total number of people participated in the trial, only 24.9% of them stated the number of participants randomized to each study group. In addition, 8.8% of abstracts mentioned about the number of participants analyzed, while merely 2.0% of abstracts described further details about the number of participants included in each experimental group. All of the abstracts described the effect estimate, and 71.9% of them were adequately reported. Most of the abstracts (96%) failed to report the confidence intervals. There were 60.5% abstracts mentioned about the P-value, and 20.5% of them reported the exact P-value. Not any of the abstracts clearly described the adoption of intention-to-treat analysis/per-protocol analysis. In addition, 37 abstracts (14.9%) adequately described adverse events.

- Reporting of trial conclusions

A total of 236 abstracts (94.8%) stated the conclusions in accordance with results. Among these abstracts, 106 abstracts (42.6%) balanced the benefits and harms.

- Analysis of associated factors with reporting quality

The results of the linear regression analysis are presented in Table 3.

**Table 3:**
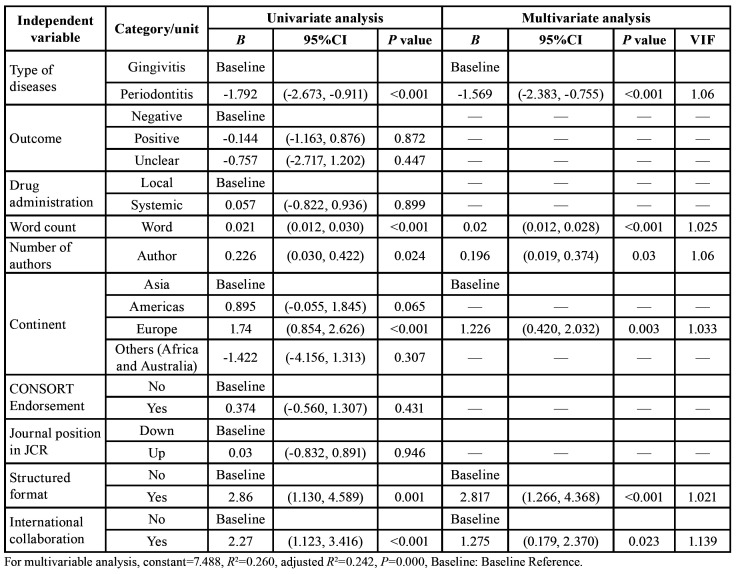
Linear regression analysis (univariate and multivariate) coefficients (<italic>B</italic>) and confidence intervals (95% CI) for quality evaluation, with the overall CONSORT score as the dependent variable (n=249).

According to the univariate analysis, abstracts that were about the gingivitis (*P*<0.001), had structured formats (*P*=0.001), higher word count (*P*<0.001) and more number of authors (*P*=0.024), had a first author that belonged to the European continent (*P*<0.001), and had international collaboration (*P*<0.001) were a significantly associated with the greater overall CONSORT score (Table 3). In the contrary, no significant differences were observed for factors as drug administration, CONSORT endorsement, journal position in JCR ranking, which were weeded out from the model. The final model included six predictors (*P*=0.000; R2=0.260 adjusted R2=0.242). Among these, type of periodontal diseases (B=-1.569; *P*<0.001), word count (B=0.020; *P*<0.001), number of authors (B=0.196; *P*=0.030), the first author from Europe (B=1.226; *P*=0.003), structured format (B=2.817; *P*<0.001) and had international collaboration (B=1.275; *P*=0.023) still persisted as a noticeable predictor of the overall CONSORT score.

## Discussion

The results of this study showed that the quality of reporting in RCTs abstracts on drug therapy of periodontal disease was suboptimal. The inadequate reporting of abstracts was in line with previous studies on dentistry (10-12), as well as on other specialties (13-15). These results manifested a lack of adherence to the CONSORT guidelines for RCT abstracts.

In terms of title, the deficient reporting of randomization may lead to irrelevant to database retrieval and omissions, leading to biases in systematic reviews. In the present study, 64.7% of abstracts titles could be identified as randomized, which was remarkably better than those of previous studies (13,16). In contrast, 13.2% of abstracts contained no information about “random”, neither in the title nor in the body of the abstract. Investigators could only identify these studies as RCTs after carefully reading their full texts. It is worth to mention that the full text could not always be easily accessed, which may lead to indexing omission by readers. In addition to mentioning “random”, the title can be improved following the PICO format (10). Surprisingly, merely 15.5% of abstracts in the present study ideally followed the PICO format.

Blinding is a very important method to reduce or even avoid bias from therapists, assessors or patients (2). The intervention design of drug therapy in periodontal RCTs can be absolutely designed as blinding. For example, Tablets used in an RCT can be made to have virtually the same appearance, so that people can’t identify the experimental Tablets from the control ones. But in our study, blinding was found to be inadequately reported. Less than half of the abstracts reported blinding, and a very few of them accurately reported who was blinded. This may result in difficulty for readers in assessing the methodological quality of RCTs through their abstracts that have not expressed blinding effectively and precisely. Additionally, bias interpretations or overestimates may be induced. A study showed that non-blinded patients might exaggerate the effect size by 0.56 standard deviation (17). Another study proved that non-blinded assessors might overestimate the effect in the treatment groups and underestimate the effect in the control groups (18).

In this study, we modified outcomes in results into three items, including effect estimate, confidence intervals, and P-value, according to a previous study published in 2013 (11). In this way, we got more details about outcomes in results. Effect estimate was well reported by a large proportion of included abstracts. But the reporting quality of confidence intervals and P-value were not so good, especially confidence intervals. These two items can reflect the statistical significance of observed treatment effects of a trial. The value of a trial in treatment decision making is whether its treatment effect can be trusted on or not (19). Poor reporting quality of confidence intervals and P-value may make it difficult for readers to assess the trial effect accurately and comprehensively. More than half of the abstracts in our study mentioned P-value, but only 20.5% of them reported the exact P-value. A study proved that approximate P-value may lead to less reliable conclusions compared to the exact P-value (20).

The CONSORT guidelines for RCT abstracts suggested reporting the funding source, especially in drug researches. It is strange to find that none of the included RCT abstracts reported “funding”. It may influence the interpretation of the trial results by readers (21). A study revealed that studies funded by pharmaceutical companies might selectively report outcomes that are more favorable to the funder when compared to researches funded by neutral sources, thereby leading to the possibility of publication bias (22). Another study also proved that the results and conclusions might more favorable for the sponsor’s drugs than the comparator ones in drug-drug comparison trials (23). So readers should be careful when reading drug RCTs to assist in their choice for drug use in clinical treatment.

We also found that six factors had a statistically significant association with reporting quality of RCT abstracts in linear regression analysis. Among them, two factors (structured format, word count) had been reported as statistically significant in the previous studies (3,12,24). It is interesting to find that abstracts on drug therapy of gingivitis were better reported than periodontitis. We also found that the vast majority of the drugs used for gingivitis therapy were contained in dentifrice or mouthwash for home dental care. So the reason for worse reporting quality in periodontitis may be that the form of drug use in periodontitis is relatively more diverse and make reporting quality unevenness. RCTs had more number of authors and international collaborations were associated with better reporting quality in abstracts. A study also found that the “number of authors” involved in conducting an RCT is directly associated with the reporting quality of the abstract (25). This may be because the authors had their own research and article submission experience. Different experiences may increase the possibility of endorsing and following the CONSORT guideline. Authors from different countries may also increase the chance to realize and follow the guidelines. Abstracts of studies conducted by European authors were better reported than authors from other continents. These findings are consistent with previous studies (8,10,12), and indicated that CONSORT guidelines were better followed by European authors.

It is delighting that an increasing number of journals stated their endorsement of the CONSORT statement in their guidelines for authors. Contrary to our expectations, although 71.9% of articles from CONSORT-endorsing journals, the reporting quality had no significant difference when compared to articles from non-endorser journals. This finding is consistent with a previous study (8). One reason for this may be the low endorsement of the CONSORT guideline for abstracts. A study in 2016 found that the endorsement of the CONSORT guideline for abstracts was only 7% (26). Another reason may be the low execution of the editors and publishers and poor compliance of authors. In consideration of the importance of RCT abstracts, as we discussed before, journals should improve the endorsement rate of CONSORT guidelines for abstracts. Editors and publishers should place more emphasis on the reporting quality of abstracts, and encourage authors to strictly adhere to the guidelines since these are especially critical in making the accelerating improvement directly.

Our study has some limitations. Firstly, our study didn’t present all the RCTs in periodontology, so our results may not be generalizable to all the RCTs in periodontology. However, drug therapy is very normal in daily clinical practice in the periodontal department and has its own features as we discussed above, so assessing their reporting quality is meaningful for clinical researchers and guideline makers. Secondly, we searched only SCI journals, some RCTs on this topic may be missed from the current research. But SCI journals are more representative than other journals. The present study has certain strengths. Firstly, it is the first time that focused on the reporting quality of drug therapy in periodontal diseases. The study comprehensively searched abstracts of 249 periodontal RCTs in 90 dental SCI journals, according to the 2018 Journal Citation Report, which differed from the previous studies, which only included several top journals in the specialty (3,10,12). The present study strictly complied with the CONSORT statement for abstracts checklist, and carefully studied its explanation in facilitating the assessment. And we added “inadequate description” and “adequate description” to score the items, and extracted more details from the included abstracts. We also summarized a checklist for the explanation of “inadequate description” and “adequate description”. A study in 2018 assessed the reporting quality of periodontal disease RCT abstracts (8), and the study just used “reported” or “no reported” to score the items. For some items, this method is enough just as funding, registration, confidence intervals. But for some items contain a lot of information, just like randomization, participants, interventions, this simple way may not be enough to assess the RCTs in a more elaborate way. For example, the vast majority of the included RCT abstracts described participants, but a few of them contained the information about where the data was collected, which was also important for this item. More details can lead us to further, and deeper analyze the reporting quality. Undoubtedly, this modified CONSORT guideline needs further study for general applicability.

## Conclusions

The reporting quality of drug therapy RCTs in periodontal disease in SCI journals was suboptimal. Clinical workers and clinical guideline makers should carefully in selecting and adopting the information in drug therapy RCTs in periodontal disease. It is not enough just to endorse the CONSORT guidelines, strictly following the guidelines to report RCT abstracts is the place where more efforts should be made.

## References

[1] Concato J, Shah N, Horwitz RI (2000). Randomized controlled trials, observational studies, and the hierarchy of research designs. N Engl J Med.

[2] Hopewell S, Clarke M, Moher D, Wager E, Middleton P, Altman DG (2008). CONSORT for reporting randomized controlled trials in journal and conference abstracts: explanation and elaboration. PLoS Med.

[3] Faggion CJ, Giannakopoulos NN (2012). Quality of reporting in abstracts of randomized controlled trials published in leading journals of periodontology and implant dentistry: a survey. J Periodontol.

[4] Chhapola V, Tiwari S, Brar R, Kanwal SK (2018). Reporting quality of trial abstracts-improved yet suboptimal: A systematic review and meta-analysis. J Evid Based Med.

[5] Hua F, Deng L, Kau CH, Jiang H, He H, Walsh T (2015). Reporting quality of randomized controlled trial abstracts: survey of leading general dental journals. J Am Dent Assoc.

[6] Needleman I, Moher D, Altman DG, Schulz KF, Moles DR, Worthington H (2008). Improving the clarity and transparency of reporting health research: a shared obligation and responsibility. J Dent Res.

[7] Leow NM, Hussain Z, Petrie A, Donos N, Needleman IG (2016). Has the quality of reporting in periodontology changed in 14 years? A systematic review. J Clin Periodontol.

[8] Kumar S, Mohammad H, Vora H, Kar K (2018). Reporting Quality of Randomized Controlled Trials of Periodontal Diseases in Journal Abstracts-A Cross-sectional Survey and Bibliometric Analysis. J Evid Based Dent Pract.

[9] Monsarrat P, Blaizot A, Kemoun P, Ravaud P, Nabet C, Sixou M (2016). Clinical research activity in periodontal medicine: a systematic mapping of trial registers. J Clin Periodontol.

[10] Fleming PS, Buckley N, Seehra J, Polychronopoulou A, Pandis N (2012). Reporting quality of abstracts of randomized controlled trials published in leading orthodontic journals from 2006 to 2011. Am J Orthod Dentofacial Orthop.

[11] Seehra J, Wright NS, Polychronopoulou A, Cobourne MT, Pandis N (2013). Reporting quality of abstracts of randomized controlled trials published in dental specialty journals. J Evid Based Dent Pract.

[12] Kiriakou J, Pandis N, Madianos P, Polychronopoulou A (2014). Assessing the reporting quality in abstracts of randomized controlled trials in leading journals of oral implantology. J Evid Based Dent Pract.

[13] Jin L, Hua F, Cao Q (2016). Reporting quality of randomized controlled trial abstracts published in leading laser medicine journals: an assessment using the CONSORT for abstracts guidelines. Lasers Med Sci.

[14] Ghimire S, Kyung E, Lee H, Kim E (2014). Oncology trial abstracts showed suboptimal improvement in reporting: a comparative before-and-after evaluation using CONSORT for Abstract guidelines. J Clin Epidemiol.

[15] Cui Q, Tian J, Song X, Yang K (2014). Does the CONSORT checklist for abstracts improve the quality of reports of randomized controlled trials on clinical pathways?. J Eval Clin Pract.

[16] Lucena C, Souza EM, Voine GC, Pulgar R, Valderrama MJ, De-Deus G (2017). A quality assessment of randomized controlled trial reports in endodontics. Int Endod J.

[17] Hrobjartsson A, Emanuelsson F, Skou TA, Hilden J, Brorson S (2014). Bias due to lack of patient blinding in clinical trials. A systematic review of trials randomizing patients to blind and nonblind sub-studies. Int J Epidemiol.

[18] Hrobjartsson A, Thomsen AS, Emanuelsson F, Tendal B, Hilden J, Boutron I (2012). Observer bias in randomised clinical trials with binary outcomes: systematic review of trials with both blinded and non-blinded outcome assessors. BMJ.

[19] Walsh M, Srinathan SK, McAuley DF, Mrkobrada M, Levine O, Ribic C (2014). The statistical significance of randomized controlled trial results is frequently fragile: a case for a Fragility Index. J Clin Epidemiol.

[20] Buas MF, Li CI, Anderson GL, Pepe MS (2018). Recommendation to use exact P-values in biomarker discovery research in place of approximate P-values. Cancer Epidemiol.

[21] Tijdink JK, Smulders YM, Bouter LM, Vinkers CH (2019). The effects of industry funding and positive outcomes in the interpretation of clinical trial results: a randomized trial among Dutch psychiatrists. BMC Med Ethics.

[22] Lexchin J, Bero LA, Djulbegovic B, Clark O (2003). Pharmaceutical industry sponsorship and research outcome and quality: systematic review. BMJ.

[23] Bero L, Oostvogel F, Bacchetti P, Lee K (2007). Factors associated with findings of published trials of drug-drug comparisons: why some statins appear more efficacious than others. PLoS Med.

[24] Song SY, Kim B, Kim I, Kim S, Kwon M, Han C (2017). Assessing reporting quality of randomized controlled trial abstracts in psychiatry: Adherence to CONSORT for abstracts: A systematic review. PLoS One.

[25] Pandis N, Polychronopoulou A, Eliades T (2010). An assessment of quality characteristics of randomised control trials published in dental journals. J Dent.

[26] Shamseer L, Hopewell S, Altman DG, Moher D, Schulz KF (2016). Update on the endorsement of CONSORT by high impact factor journals: a survey of journal "Instructions to Authors" in 2014. Trials.

